# Proteomic and metabolomic profiling reveals the underlying molecular mechanisms in modified alternate-day fasting-mediated protection against Diabetic kidney disease

**DOI:** 10.1371/journal.pone.0319053

**Published:** 2025-02-18

**Authors:** Xin Zeng, Yi-hang Xing, Xiu-mei Ma, Yang Long, Zong-zhe Jiang, Yong Xu

**Affiliations:** 1 Department of Endocrinology and Metabolism, The Affiliated Hospital of Southwest Medical University, Luzhou, Sichuan, People’s Republic of China; 2 Sichuan Provincial Key Laboratory for Human Disease Gene Study and Department of Laboratory Medicine, Sichuan Provincial People’s Hospital, University of Electronic Science and Technology of China, Chengdu, China; 3 Metabolic Vascular Disease Key Laboratory of Sichuan Province, Sichuan, People’s Republic of China; 4 Sichuan Clinical Research Center for Nephropathy, Luzhou, Sichuan, People’s Republic of China; Neyshabur University of Medical Sciences, IRAN, ISLAMIC REPUBLIC OF

## Abstract

**Background:**

Diabetic kidney disease (DKD) is a leading cause of chronic kidney disease, and while lifestyle interventions like intermittent fasting have shown promise in treating diabetes, the impact of modified alternate-day fasting (MADF) on DKD is not well understood. This study aimed to explore MADF’s effects on DKD in db/db mice, a model for the condition, and to investigate its underlying mechanisms.

**Methods:**

We implemented an MADF regimen in db/db mice on a high-fat diet, measuring blood glucose, body weight, and renal function at various times. After the intervention, we analyzed the proteome and metabolome of renal tissues.

**Results:**

MADF was found to reduce hyperglycemia and slow the pathological progression of DKD in the mice. Proteomic analysis identified 165 proteins that increased and 196 that decreased in the kidneys of db/db mice compared to controls. MADF intervention led to a decrease in 26 of the increased proteins and an increase in 18 of the decreased ones. Notably, many of these proteins, including cathepsin S (CTSS), were related to lysosomes, suggesting a role in renal protection. Metabolomic profiling revealed changes in metabolites associated with inflammation, such as prostaglandin A1, which was downregulated in db/db mice and upregulated with MADF. Western blotting, immunohistochemistry, and immunofluorescence staining confirmed the expression changes of CTSS observed in the proteomic data. Additionally, CTSS expression was found to increase in renal cells exposed to high glucose and palmitic acid.

**Conclusion:**

MADF appears to mitigate the progression of DKD, with proteomic evidence pointing to lysosome-related proteins like CTSS as potential mediators of its renal protective effects. These findings indicate that MADF and the inhibition of CTSS could be considered as novel therapeutic strategies for DKD treatment.

## Introduction

Diabetes is one of the most prevalent noncommunicable diseases worldwide. The number of individuals suffering from diabetes rose by 74 million in 2019 and reached 537 million globally in 2021. The International Diabetes Federation speculates that the figure will escalate to 783 million by 2045, making a 46% rise, which surpasses the anticipated population expansion by over twofold during that timeframe [1]. Type 2 diabetes mellitus (T2DM) accounts for over 90% of the entire diabetic population [2]. The danger of diabetes primarily lies in its various complications. Diabetic kidney disease (DKD) is one of the most common microangiopathies associated with T2DMand is the leading cause of end-stage renal disease. In previous studies, DKD was also referred to as diabetic nephropathy (DN), both indicating renal complications arising from diabetes mellitus. However, the KDIGO guidelines recommend using DKD instead of DN [3]. Although these two terms are often used interchangeably, DN typically refers to the results of renal pathological biopsy, which is now less commonly used; whereas DKD refers to chronic kidney disease (CKD) that occurs in the context of diabetes without other contributing factors, such as hypertension or autoimmune diseases [4]. In this study, CKD that occurs in the context of diabetes is uniformly denoted by DKD. DKD is characterized by a persistent increase in albumin excretion and a progressive decrease in the glomerular filtration rate, ultimately leading to the development of end-stage renal disease [5,6]. Globally, approximately 30–50% of all cases of end-stage renal disease is caused by DKD. In China, DKD has become the primary cause of end-stage renal disease among middle-aged and older adults [7]. Therefore, DKD jeopardizes the population’s health and seriously affects socioeconomic development. Currently, preventive measures and treatments for DKD have limited benefits in terms of reducing morbidity and mortality rates.

The prevention and treatment of DKD mainly focus on strict and effective glycemic and blood pressure control. In recent years, more and more studies have shown that nephroprotective drugs, such as renin–angiotensin–aldosterone system inhibitors [8,9], mineralocorticoid receptor antagonist [10–12], sodium-glucose cotransporter-2 inhibitors [13,14], and glucagon-like peptide 1 agonists [15], can effectively reduce proteinuria, improve kidney function and delay the progression of DKD. However, these medications are mainly used to treat DKD in patients with T2DM and may not be generalizable to type 1 diabetes without additional clinical evidence. Nevertheless, despite the use of these drugs, the complex physiological mechanisms of DKD make its progression unavoidable [16,17].

Dietary restriction is a reliable and effective nonpharmacological intervention for several metabolic diseases. Currently, primary dietary restriction programs include calorie restriction, intermittent fasting, restriction of specific macronutrients, ketogenic diet, and fasting-mimicking diet [18]. Dietary restriction reduces the body weight, improves the metabolic and health status, and increases the lifespan of people [19–21]. Recently, intermittent fasting has become increasingly popular, with more choices for intermittent fasting modalities. Established clinical studies have confirmed that the weight loss and metabolic improvement effects of intermittent fasting and calorie restriction diets are similar [22–25]. Intermittent fasting reduces body weight, lowers fasting insulin levels, increases insulin sensitivity, decreases insulin resistance lowers postprandial glucose levels, and improves blood pressure and lipid levels, implying that intermittent fasting affects metabolic improvement and cardiovascular protection [26–28]. Thus, intermittent fasting is favored as an alternative to calorie restriction and may be a practical dietary approach for improving metabolic syndrome and diabetes.

The main types of intermittent fasting include time-restricted feeding/eating (TRF/TRE), 5:2 fasting (5:2 diet), and alternate-day fasting (ADF). Since 2018, several studies have been conducted on ADF and TRF/TRE [21], with ADF having been reported to lead to better weight loss and compliance than TRF/TRE and 5:2 fasting [29,30]. ADF is composed of an *ad libitum* day (on which no restriction is placed on the time and amount of food consumed) and a fasting day (on which calorie intake is entirely restricted and no restriction is placed on the intake of calorie-free water [zero-calorie ADF] or on which the intake of 20–30% of the usual amount of food consumed is allowed [modified ADF] (MADF)) [29]. Modified fasting on alternate days has been shown by clinical studies to improve lipid metabolism and to reduce hepatic lipid deposition in patients with nonalcoholic fatty liver disease [31]. It also improves blood pressure and blood glucose levels and contributes to cardiovascular protection [32]. A previous study comparing the effect of MADF on DKD using a mouse model of type 2 DKD revealed that MADF reduced the renal weight and 24-h urine output, improved DKD, and delayed its progression. Nonetheless, the possible molecular mechanism remains unclear [33].

Previous study reported that DNA sequence across the entire body can be regarded as static and uniform considering kidney disease [34]. While the genome is static, the proteome and metabolome are dynamic and can provide real-time insights into the biological processes occurring within the kidney. These analyses are crucial for understanding the pathophysiology of DKD, as they can capture the variable molecular responses to the disease [35]. Proteomic and metabolomic approaches allow for the identification of biomarkers that can predict the onset and progression of DKD, facilitating early intervention strategies [36,37]. For instance, Han et al. analyzed plasma samples from patients at different stages of DKD and found that the concentration of non-esterified fatty acids (NEFAs) increased, while the concentration of esterified fatty acids (EFAs) decreased in the early stages of DKD [38]. High concentrations of free fatty acids play a major role in endothelial cell damage and dysfunction [39]. The changes in NEFAs and EFAs reported by Han et al. are considered metabolic adjustments in response to kidney disease progression, ultimately leading to varying degrees of organ damage.

Diabetic C57BLKS-Lepr^db/db^ (db/db) mice, which are leptin receptor-deficient, serve as a prominent model due to their development of obesity, hyperglycemia, dyslipidemia, and insulin resistance, making it a well-established representation of T2DM [40]. The db/db mice exhibit a pronounced DKD phenotype characterized by albuminuria and significant histological changes, including an increase in the thickness of the glomerular basement membrane, a reduction in podocyte numbers [41], and a moderate degree of mesangial expansion [42]. Non-diabetic C57BLKS-Lepr^db/+^ (db/m) mice, which are heterozygous for the leptin receptor mutation, share the same genetic background with db/db mice, originating from the C57BLKS/J strain [43]. Due to their phenotypic similarities to wild-type mice, db/m mice are commonly used as a control group in experiments.

In this study, we established a type 2 DKD model using high-fat diet (HFD)-fed db/db mice to investigate the effects of ADF on DKD and to explore its potential molecular mechanisms. We employed a combined proteomic and metabolomic linkage analysis approach to provide a comprehensive understanding of the molecular mechanisms by which ADF could ameliorate DKD. This integrated strategy aims to enhance our understanding of DKD pathophysiology and to identify potential therapeutic targets, potentially leading to the development of novel therapeutic strategies for DKD management.

## Methods

### Animals

This study employed 16 5-week-old male db/db mice and 8 5-week-old male db/m mice, sourced from GemPharmatech Co., Ltd., Nanjing, China. The db/db mice were randomly allocated into two distinct cohorts: one receiving a high-fat diet alone (HFD group, n =  8) and the other receiving the same diet combined with modified ADF (HFD +  MADF group, n =  8). The mice in these two groups were fed with an HFD (60% fat). The control group consisted of db/m mice that were provided a standard diet (SD group, n =  8). Mice in the HFD group were provided with adequate food without restrictions on the amount and duration of feeding, whereas mice in the HFD +  MADF group were subjected to MADF dietary intervention, in which adequate food was provided on feeding days and only one-third of the food consumed on the previous day was provided on fasting days. All mice were kept in individually ventilated cages, and subjected to a12-h alternating light and dark schedule, at a constant temperature of 23 ±  1°C and a relative humidity of 60 ±  10%. Every two weeks, the mice were assessed for changes in weight and blood glucose levels. All mice were euthanized after 12 weeks of dietary intervention using a combination of intraperitoneal injection of pentobarbital sodium followed by cervical dislocation. All procedures were conducted by trained personnel to minimize any potential suffering. The animal experiments were permitted by the Animal Ethics Committee at Southwest Medical University (NO.20220304-003) and complied with the ethical principles for the utilization of laboratory animals as outlined by the National Institutes of Health.

### Cell culture

The human renal proximal tubular epithelial cells (HK-2), acquired from ATCC in the USA, were propagated in DMEM/F12 (Invitrogen, Carlsbad, CA, USA) with the addition of 10% fetal bovine serum (ScienCell Research Laboratories, USA) and 1% penicillin-streptomycin solution (Beyotime, Shanghai, China), under condition of 37°C temperature and 5% CO_2_. High glucose (30 mmol/L) and palmitic acid (100 μmol/L) (HGPA) were added to the HK-2 cell medium for 24 h to detect the expression of specific proteins.

### Urinary protein assessment

Urinary Albumin-to-Creatinine Ratio (UACR) is recognized as a renal injury marker and an early indicator of diabetic kidney pathology. Typically, UACR becomes elevated before a decline in estimated glomerular filtration rate, making it a crucial tool for the early detection of DKD [44]. In our study, the levels of urinary protein and creatinine in the mice were analyzed with kits (Nanjing Jiancheng Bioengineering Institute, China). Detection was performed using the enzyme-linked immunosorbent assay technique in strict adherence to the guidelines given by the kit’s producer.

### Histopathological examination

The evaluation of histopathological specimens was performed following the techniques that were previously reported [45]. Briefly, renal tissue samples were first fixed in 4% paraformaldehyde, embedded in paraffin, and cut into 4-μm-thick sections to facilitate detailed tissue analysis. Morphometric assessments were performed with hematoxylin and eosin (H&E) staining. Renal fibrosis was visualized using Masson’s trichrome staining.

### Immunohistochemistry staining

To assess renal injury and inflammation, we selected kidney injury molecule-1 (KIM-1), interleukin-18 (IL-18), interleukin-1β (IL-1β) as markers. KIM-1 is a transmembrane glycoprotein that is upregulated in the renal proximal tubular epithelial cells following kidney injury, making it an early urinary biomarker for kidney tubular damage [46]. IL-18 and IL-1β are cytokines known to mediate inflammation, a key component of DKD pathogenesis [47].

The renal tissue was preserved by fixation in 4% paraformaldehyde, encased in paraffin, and sectioned to a thickness of 4μm. Renal tissues were dewaxed by immersion in xylene and subsequent rehydration using an ethanol gradient. An immunohistochemical (IHC) kit (abs957, Absin Bioscience, Shanghai, China) was used to identify the expression of KIM-1, IL-18, IL-1β and cathepsin S (CTSS) in the kidney of mice according to the manufacturer’s instructions. For IHC staining, the first antibodies used were anti-KIM-1 (1:100, ab78494, Abcam), anti-IL-18 (1:100, AF5207, Beyotime), anti-IL-1β (1:100, AF7209, Beyotime), and anti-CTSS (1:100, ab232740, Abcam). The stained slides were examined with a microscope.

### TdT-mediated dUTP nick end labeling (TUNEL) assay

TUNEL staining was employed to evaluate cellular apoptosis within the renal tissue, providing insight into the cellular mechanisms contributing to DKD progression [46]. Apoptotic activity in paraffin-embedded renal tissue sections was quantitatively assessed by detecting DNA fragmentation using TUNEL BrightRed Apoptosis Detection Kit (A113-02, Vazyme, Nanjing, China) based on the manufacturer’s instruction. In brief, the renal sections were gently washed with phosphate-buffered saline (PBS) and then incubated in TdT buffer for 20 minutes Subsequently, the TdT buffer was carefully removed, and a terminal transferase reaction mixture was added, which was permitted to react for one hour at 37°C. The sections were then washed with PBS, and all renal tissue samples from each section were examined under a fluorescence microscope (Leica, Germany). The nuclei of TUNEL-positive cells emanated red fluorescence.

### Immunofluorescence staining

For immunofluorescence staining of HK-2 cells, paraffin-embedded sections were stained with an anti-CTSS antibody (1:100, ab-232740, Abcam). Incubation with Cy2/FITC dye-tagged secondary antibodies (1:200, Biosynthesis Biotech, China) was carried out in darkness for 60 minutes. The nuclei were then stained with DAPI, and micrographs were recorded on a Leica microscope.

### Western blot analysis

Proteins were extracted from HK-2 cells and mouse renal tissue samples using a lysis buffer composed of radioimmunoprecipitation assay solution (Beyotime) supplemented with 1% protease inhibitor (Beyotime). The extracted proteins were fractionated through SDS–polyacrylamide gel electrophoresis onto PVDF membranes (Millipore, MA, USA). The membranes were soaked with a 5% bovine serum albumin and anti-CTSS antibody (1:1000, ab232740, Abcam). After extensive washing, the membranes were incubated with species-specific horseradish peroxidase-conjugated secondary antibodies (goat anti-mouse or anti-rabbit IgG) tailored to the primary antibodies. Immunoreactive bands were detected using an enhanced chemiluminescence system (Millipore). The intensity of protein bands was quantified using Image J software.

### Proteomic analysis

#### Tissue samples.

Samples were ground into powder with the aid of liquid nitrogen and placed into a 5-mL centrifuge tube. Subsequently, lysis buffer (8 M urea, 1% protease inhibitor cocktail) in a fourfold volume was added to the powder, followed by 3 minutes of sonication on ice with an ultrasonic cell disruptor (Scientz, Hangzhou, China). The residual material was collected as a pellet via centrifugal force at 12,000 gravitational force units for a period of 10 minutes at a temperature of 4°C, and the clear liquid was extracted. The protein content was measured using a BCA protein quantification kit.

#### Trypsin digestion.

Samples were gradually introduced to attain a final volume percentage of 20% (m/v) trichloroacetic acid to cause protein sedimentation, followed by vigorous shaking and a 2-hour incubation period at a temperature of 4°C. The resulting precipitate was gathered through centrifugal force at 4500 g for 5 minutes at a chill temperature of 4°C. The sedimented proteins were cleansed three times using chilled acetone and then briefly dried for a minute. Subsequently, 300μL of 200mM tetraethylammonium bromide solution was added to the protein extracts and subjected to ultrasonic wave dispersion. The protein extracts were first treated with a reduction agent, 1.5μL of 1 mM dithiothreitol (DTT), for 60 minutes at 37°C to cleave disulfide bonds. Following reduction, the samples were alkylated using 6.15μL of 550mM iodoacetamide for 45 minutes in the dark to prevent reformation of disulfide bonds. After the DTT and iodoacetamide reactions, the proteins were then subjected to enzymatic digestion with trypsin overnight at a mass ratio of trypsin to protein of 1:50. After tryptic digestion, the peptides were desalted using a Strata X SPE column, during which the DTT and IAM are also removed.

#### Proteomic analysis.

For the proteomic analysis, we first selected the samples that required comparison. The ratio of the mean relative quantitative values of each protein across multiple replicate samples was determined as the fold change (FC). To assess the significance of the differences, we performed a T-test on the relative quantitative values of each protein in the compared groups and calculated the corresponding P values, using a P value of less than 0.05 as the threshold for significance. Prior to T-testing, to ensure that the data met the normality requirement for T-testing, we performed a Log2 transformation on the relative quantitative values of the proteins. Through the aforementioned differential analysis, we considered a P value less than 0.05 and a change in expression greater than 1.5-fold to indicate significant upregulation, and less than 1.5-fold to indicate significant downregulation. Additionally, we constructed a volcano plot for the compared groups with P values, where the x-axis represents the Log2-transformed fold change values and the y-axis represents the -Log10-transformed P values. Furthermore, we generated a heatmap for the union of all differential proteins from the compared groups to display the relative expression levels of multiple proteins across different samples, revealing the clustering of the relative expression levels of the differential proteins. We also conducted Gene Ontology (GO) analysis and Kyoto Encyclopedia of Genes and Genomes (KEGG) pathway classification analysis for the differential proteins identified.

### Metabolomic analyses

First, 15 mg of tissues was homogenized with 150 μL of H_2_O, and 600 μL of MeOH/ACN (1:1, v/v) solvent mixture was then mixed with the homogenized tissue samples. The samples were vortexed for 30 seconds, followed by sonication for 10 minutes on ice using a high-intensity ultrasonic processor. Subsequently, the specimens were subjected to rapid freezing in liquid nitrogen for 1 minute, followed by a complete thawing phase at ambient temperature. Then, they were treated with sonication for 3 minutes while being kept on a chilled surface, utilizing a high-power ultrasonication device. These aforementioned steps were repeated three times. Afterward, the specimens were held at -20°C for 1 hour, and the leftover residue was eliminated through the process of centrifugation at a force of 12,000 g for 15 minutes at a temperature of 4°C. The supernatant was then gathered and reduced to a dry state by employing a vacuum evaporator at ambient temperature. Subsequently, 150 μL of ACN/H_2_O (1:1, v/v) solvent mixture was added to the dried samples. The specimens were agitated for 30 seconds using a vortex mixer and then treated with a high-intensity ultrasonic device on ice for 10 minutes. The remaining debris was cleared by centrifugation at a rate of 18,000 g for 15 minutes at 4°C. Ultimately, the supernatant was kept at -80°C for subsequent LC/MS analysis.

Peptides were dissolved in solvent A of the liquid chromatography mobile phase and then separated using a NanoElute ultra-high-performance liquid chromatography (UHPLC) system. Mobile phase A consisted of an aqueous solution containing 0.1% formic acid and 2% acetonitrile; mobile phase B was an acetonitrile-water solution containing 0.1% formic acid. The liquid chromatography gradient was set as follows: 0-70 min, 6% to 24% B; 70.0-84.0 min, 24% to 35% B; 84.0-87.0 min, 35% to 80% B; 87.0-90.0 min, 80% B, with a flow rate maintained at 450 nl/min. After separation by the UHPLC system, peptides were injected into a Capillary ion source for ionization and then introduced into a timsTOF Pro (Bruker) mass spectrometer for analysis. The ion source voltage was set at 2.0 kV, and both parent ions and their product ions were detected and analyzed using high-resolution time-of-flight (TOF). Data acquisition was performed using the Parallel Accumulation Serial Fragmentation (PASEF) mode. After one full MS1 spectrum acquisition, 10 PASEF acquisitions of product ion spectra with parent ion charges ranging from 0 to 5 were performed, and the dynamic exclusion time for the MS/MS scan was set to 30 seconds to prevent repeated scanning of parent ions.

### Statistical analysis

Data from Masson’s trichrome staining, IL-18, IL-1β, KIM-1, TUNEL staining, tissue WB, and cellular WB were expressed as means ±  standard deviations. Statistical analyses were performed using GraphPad Prism 10 and SPSS 22.0 software. The normality of all data sets was assessed with Shapiro-Wilk’s test. Student’s *t*-test was conducted to evaluate the differences between two independent groups, with the assumption of normality and equal variances in the data sets. One-way analysis of variance (ANOVA) and Tukey’s HSD test were used to assess the differences among multiple groups for data with normally distribution and unequal variances. For data with normally distribution and unequal variances, Welch’s ANOVA was employed to assess overall differences among groups, followed by Dunnett’s T3 multiple comparisons test to identify specific pairwise differences. Statistical significance was established at the P <  0.05 threshold (*P <  0.05, **0.001 <  *P* <  0.01, and ***P <  0.001).

## Results

### MADF reversed diabetes-induced renal injury in db/db mice

**Fig 1:**
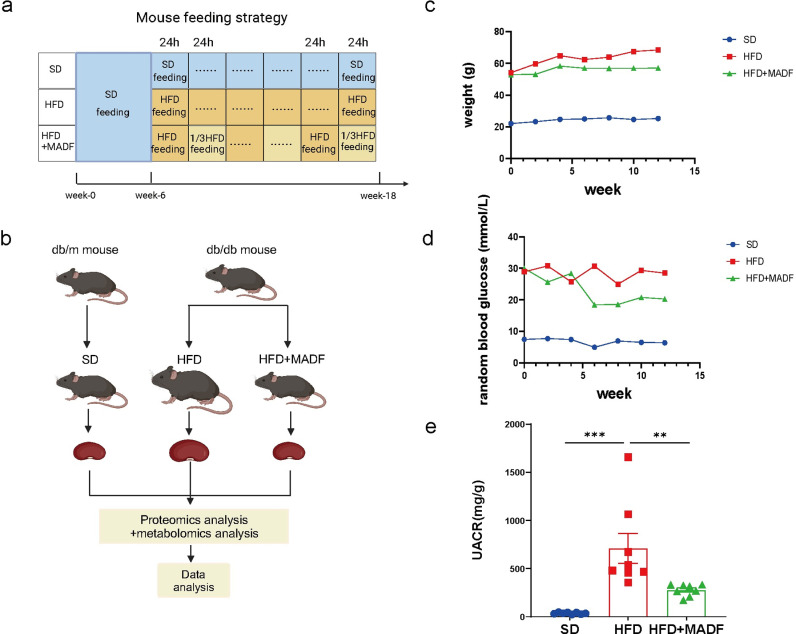
MADF improved the body weight, blood glucose, and renal function in db/db mice. The dietary intervention protocol used in this study. (b) Experimental procedure. (c) Body weight changes in mice during the experiment. (d) Random blood glucose changes in mice during the experiment. (e) Urinary albumin-to-creatinine ratio in the three mouse groups at the end of the intervention (n =  8 in each group). One-way ANOVA and Tukey’s HSD test were used to analyze the data in (e).

**Fig 2:**
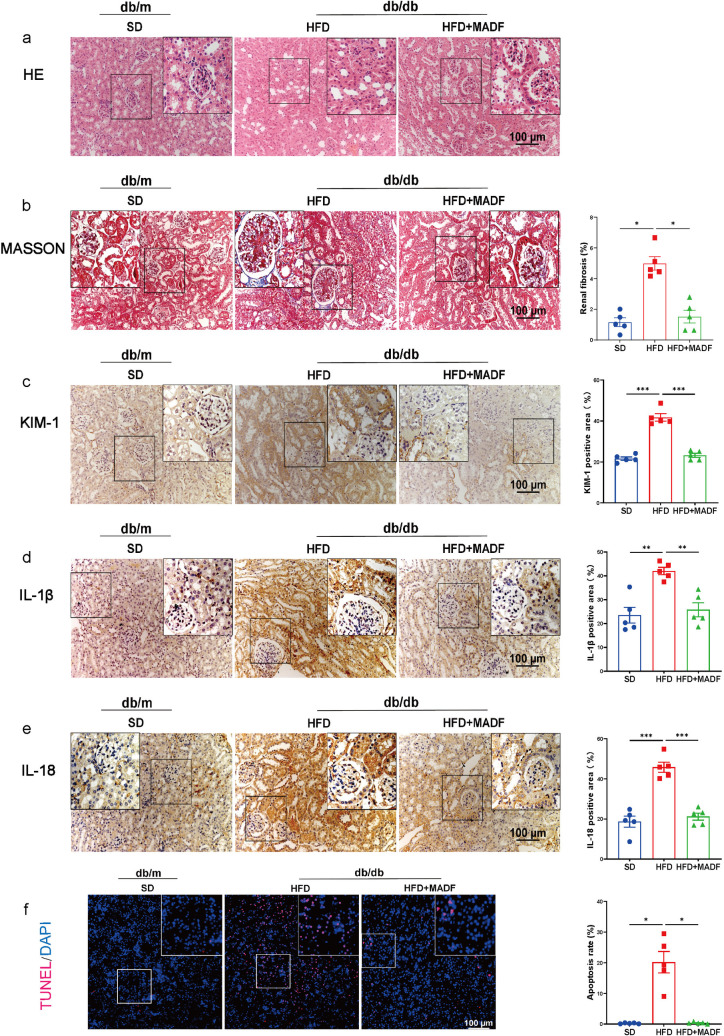
MADF attenuated renal injury in db/db mice. (a) Representative H&E staining images showing pathological changes in the kidneys. (b) Representative Masson’s trichrome staining images showing renal fibrosis (*n* =  5 per group). (c and d) Representative immunohistochemical photomicrographs (IL-1β, IL-18, and KIM-1) showing the pathological alterations in the kidneys in the indicated groups (*n* =  5 per group). One-way ANOVA and Tukey’s HSD test were used to analyze the data in (b), (c), and (d). (e) Apoptosis in the kidneys (*n* =  5 per group). Welch’s ANOVA and Dunnett’s T3 test was used to analyze the data in (e). Data are expressed as means ±  standard deviations. * *P* <  0.05, **P <  0.01, ***P <  0.001. HFD: high-fat diet group, SD: control group, HFD +  MADF: high-fat group with alternate-day fasting intervention.

To verify whether MADF had an ameliorative effect on DKD, we constructed a DKD model using db/db mice as the experimental group and db/m mice as the control group and implemented a 12-week MADF dietary intervention (Fig 1a–1b). With the intervention, changes in body weight and blood glucose levels were detected among the mice. As illustrated in Fig 1c and S1 Fig, the rate of increase of weight is lower for HFD+MADF group over HFD group. Compared to HFD group, the group that received a HFD combined with MADF exhibited reduced average blood glucose levels (Fig 1d). The UACR was measured in all mice at the end of the intervention (Fig 1e). One-way ANOVA revealed significant differences among the groups, with a F-ratio of 14.21 and a *P* value of 0.001, indicating that there was a statistically significant difference between at least two of the three groups. Tukey’s HSD test identified significant differences between the SD group and the HFD group (mean difference (MD) =  -675.7, 95% confidence interval (CI) =  -999.4 to -352.0, *P* <  0.001, t-statistic (difference of two means over standard error) =  -5.26) and between HFD group and HFD +  MADF group (MD =  433.6, 95% CI =  109.9 to 757.3, *P* =  0.0077, t-statistic =  3.38), but bot between the SD group and the HFD +  MADF group (MD =  -242.1, 95% CI =  -565.8 to 81.58, *P* =  0.1677, t-statistic =  -1,89). This result indicated the potential therapeutic effect of MADF in ameliorating renal function in DKD mice. With respect to the histological analysis, H&E staining revealed that the DKD mice undergoing the MADF intervention exhibited normal glomerular morphology, in contrast to the glomerular hypertrophy observed in DKD mice not undergoing the MADF intervention (Fig 2a). Furthermore, MADF led to a noticeable attenuation of renal fibrosis in DKD mice (Fig 2b), with one-way ANOVA confirming significant group difference (F =  14.21, *P* =  0.001). Tukey’s HSD test highlighted significant effects: SD vs. HFD (MD =  -3.843, 95% CI =  -5.331 to -2.374, *P* <  0.001, t-statistic =  -6.98); HFD vs. HFD +  MADF (MD =  3.473, 95% CI =  2.005 to 4.941, *P* =  0.001, t-statistic =  6.31). No significant difference was observed between SD group and HFD +  MADF group (MD =  -0.3695, 95% CI =  -1.838 to 1.099, *P* =  0.78, t-statistic =  -0.67). Importantly, the immunohistochemical analysis indicated increased KIM-1 expression in the renal tissues of DKD mice, suggesting renal damage. However, MADF substantially reduced the KIM-1 levels (Fig 2c), highlighting the protective effects of this intervention (one-way ANOVA: F =  76.83, *P* <  0.001. Tukey’s HSD test: SD vs. HFD, MD =  -20.15, 95% CI =  -24.98 to -15.33, *P* <  0.001, t-statistic =  -11.14; SD vs. HFD +  MADF, MD =  -1.555, 95% CI =  -6.382 to 3.271, *P* =  0.675, t-statistic =  -0.86; HFD vs. HFD +  MADF, MD =  18.60, 95% CI =  13.77 to 23.42, *P* <  0.001, t-statistic =  10.28). MADF also led to a significant decrease in the levels of IL-18 and IL-1β, both of which were associated with DKD pathology (Fig 2d–2e) (IL-18: one-way ANOVA: F =  39.72, *P* <  0.001. Tukey’s HSD test: SD vs. HFD, MD =  -27.14, 95% CI =  -36.12 to -10.17, *P* <  0.001, t-statistic =  -8.07; SD vs. HFD +  MADF, MD =  -2.519, 95% CI =  -11.50 to 6.459, *P* =  0.74, t-statistic =  -0.75; HFD vs. HFD +  MADF, MD =  24.62, 95% CI =  15.65 to 33.60, *P* <  0.001, t-statistic =  7.32) (IL-1β: one-way ANOVA: F =  13.90, *P* =  0.001. Tukey’s HSD test: SD vs. HFD, MD =  -18.52, 95% CI =  -28.74 to -8.299, *P* =  0.0011, t-statistic =  -4.83; SD vs. HFD +  MADF, MD =  -2.278, 95% CI =  -12.5 to 7.493, *P* =  0.8255, t-statistic =  -0.59; HFD vs. HFD +  MADF, MD =  16.24, 95% CI =  6.020 to 26.46, *P* =  0.003, t-statistic =  4.24). Additionally, the TUNEL assay showed the mitigating effect of MADF on apoptosis in the kidneys of DKD mice, suggesting its potential role in preserving renal cell viability (Fig 2f) (Welch’s ANOVA: F =  15.06, *P* =  0.0035. Dunnett’s T3 test: SD vs. HFD, MD =  -19.96, 95% CI =  -33.06 to -6.850, *P* =  0.0119, t-statistic =  -5.7; SD vs. HFD +  MADF, MD =  -0.1331, 95% CI =  -0.6161 to 0.3499, *P* =  0.7661, t-statistic =  0.88; HFD vs. HFD +  MADF, MD =  19.82, 95% CI =  6.711 to 32.94, *P* =  0.0122, t-statistic =  5.66). Collectively, these findings underscored the therapeutic potential of MADF in ameliorating DKD by suppressing inflammation, reducing kidney injury marker levels, and inhibiting cell death.

### Proteomic profiling revealed differentially expressed proteins in the kidneys of db/db mice after the MADF intervention

**Fig 3:**
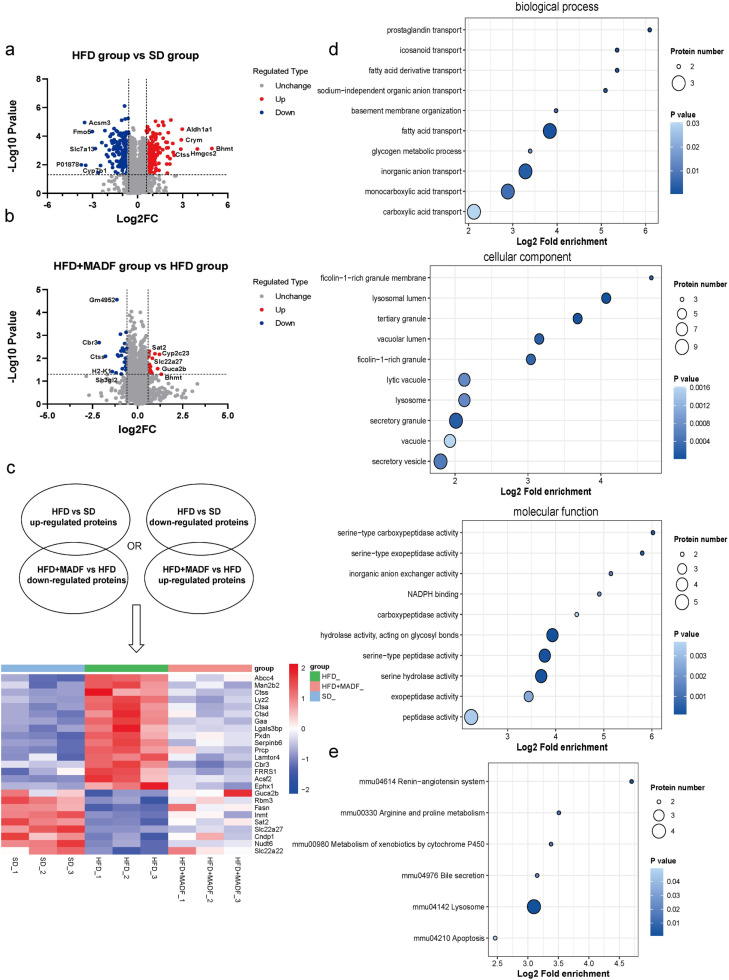
Quantitative analysis of differentially expressed proteins in mouse renal tissues. (a) Volcano plot of differentially expressed proteins in the HFD group compared with the SD group. (b) Quantitative volcano plot of differentially expressed proteins in the HFD group compared with the HDF +  MADF group. Gray denotes the expression of non-significantly different proteins in the two groups, red indicates the expression of upregulated proteins, and blue represents the expression of downregulated proteins. (c) Heatmap of differentially expressed proteins in the mouse renal tissues, with red and blue in the heatmap indicating higher and lower protein expression, respectively. (d) Three functional categories in the Gene Ontology (GO) analysis: Biological Process, Cellular Component, and Molecular Function. The size and color of bubbles indicate the number of proteins contained in the pathway and the *P*-value, respectively. (e) Kyoto Encyclopedia of Genes and Genomes (KEGG) pathway enrichment analysis. The size and color of bubbles indicate the number of proteins contained in the pathway and the *P*-value, respectively. HFD: high-fat diet group; SD: control group; HFD +  MADF: high-fat group with alternate-day fasting intervention.

We performed a proteomic analysis on the mouse renal tissues to explore the potential mechanisms underlying the ameliorative effect of MADF on DKD. A total of 4,330 proteins were measured in the kidneys of db/db mice in the HFD group versus the SD counterparts. Overall, 361 proteins exhibited significant expression alterations, deemed significant with a P-value below 0.05 and fold changes exceeding 1.5 or falling below -1.5. This cohort comprised 165 upregulated proteins and 196 downregulated proteins (Fig 3a and S1 Table). A subset of 44 proteins showed substantial modifications in the kidneys of DKD mice following the MADF intervention. Specifically, this subset consisted of 18 upregulated proteins and 26 downregulated proteins (Fig 3b and S2 Table).

Subsequently, we focused on a selected group of proteins exhibiting contrasting patterns of alterations and subjected these meticulously chosen proteins to advanced analyses, including heatmap, GO, and KEGG pathway analyses. Fig 3c shows a heatmap of 25 distinct proteins.

GO and KEGG pathway enrichment analyses were conducted, and the top ten pathways were selected to draw bubble diagrams (Fig 3d–3e). The GO analysis of biological processes revealed that these proteins were mainly involved in the transport of prostaglandin, icosanoid, and fatty acid derivatives. Remarkably, the primary genes involved in these pathways were *Abcc4* and *Slc22a22*, highlighting their critical involvement in the regulation of intricate mechanisms of lipid metabolism and signaling. With respect to cellular components, these differentially expressed proteins were notably enriched in ficolin-1-rich granule membranes, in which *Gaa*, *Serpinb6*, *Prcp*, *Ctss*, and *Ctsd* genes were involved, and in the lysosomal lumen, in which *Man2b2*, *Gaa*, *Ctsa*, *Ctsd*, and *Ctss* genes were involved. In terms of molecular function, these proteins were mainly enriched in serine-type carboxypeptidase activity and serine-type exopeptidase activity, and the genes involved were *Prcp* and *Ctsa*, underscoring their importance in the regulation of peptide processing and metabolism. KEGG pathway analysis revealed that the ameliorative effect of MADF on DKD mainly involved the renin–angiotensin system, arginine and proline metabolism, xenobiotic metabolism by cytochrome P450, bile secretion, lysosomes, and apoptosis. We found that genes exhibiting differential expression were mostly related to the lysosomal pathway on the GO analysis and were enriched in lysosomes on the KEGG pathway enrichment analysis. Among the differentially expressed proteins identified through screening, CTSS, cathepsin D (CTSD), and cathepsin A (CTSA) were most closely related to lysosomes.

### Metabolomic profiling revealed differentially expressed metabolites in the kidneys of db/db mice after the MADF intervention

**Fig 4:**
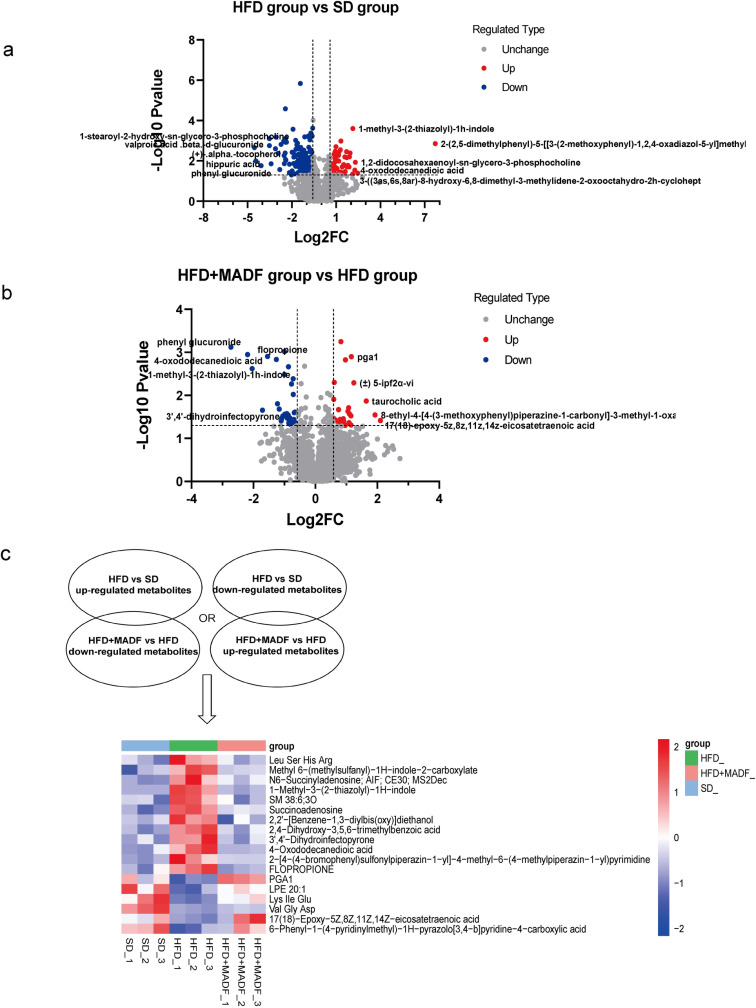
Quantitative analysis of differentially expressed metabolites in mouse renal tissues. (a) Volcano plot of differentially expressed metabolites in the HFD group compared with the SD group. (b) Volcano plot of differentially expressed metabolites in the HFD group compared with the HFD +  MADF group. Gray denotes the expression of non-significantly different metabolites in the two groups, red indicates the expression of upregulated metabolites, and blue represents the expression of downregulated metabolites. (c) Heatmap of differentially expressed metabolites in mouse renal tissues, with red and blue in the heatmap denoting the expression of higher and lower metabolites, respectively. HFD: high-fat diet group, SD: control group, HFD +  MADF: high-fat group with alternate-day fasting intervention.

We detected 2,100 metabolites in the HFD group compared with the SD group, with a total of 259 differentially expressed metabolites. Among these metabolites, 63 metabolites were upregulated, whereas 196 metabolites were downregulated (Fig 4a and S3 Table).

We also detected 2,100 metabolites in the HFD +  MADF group compared with the HFD group, with a total of 57 differentially expressed metabolites (*P* <  0.05, FC >  1.5 or FC < -1.5). Among these metabolites, 25 metabolites were upregulated, whereas 32 metabolites were downregulated (Fig 4b and S4 Table).

As shown in Fig 4c, we showed the differential metabolites using a heatmap. The levels of anti-inflammatory metabolites and lipid metabolism-regulatory compounds, such as prostaglandin A1, 5-ipfza-vi, and taurocholic acid, increased after the MADF intervention in DKD mice. Notably, the levels of the vasoactive agent 3’,4’-dihydroinfectopyrone decreased. Moreover, the levels of fatty acid derivative 4-oxododecanedioic acid and the antispasmodic agent flopropione also decreased.

### Integrated analysis of proteomic and metabolomic data

**Fig 5:**
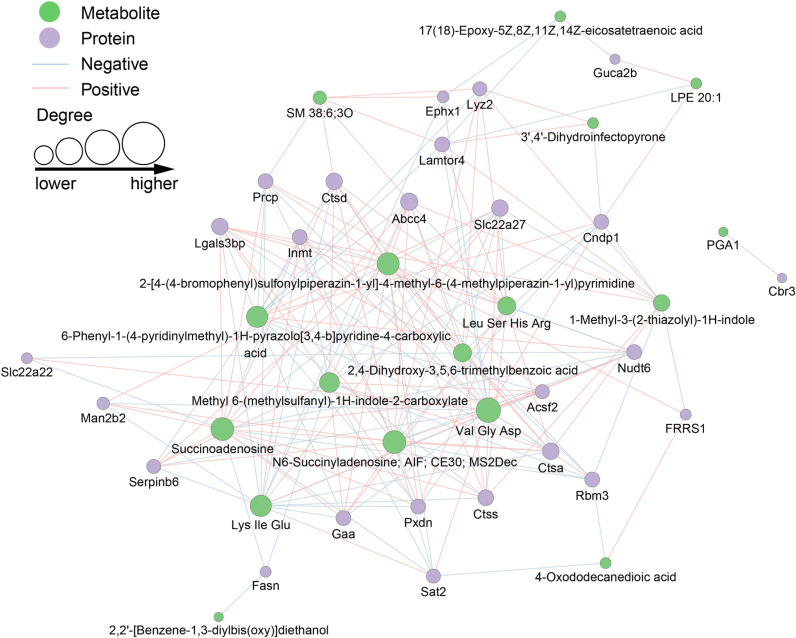
Mouse renal tissues differentially expressed protein–metabolite co-expression networks. The dots represent the proteins and metabolites, and the connecting lines between them denote their correlations.

For the integrated analysis, we used the R programming language to calculate Spearman correlation coefficients between protein expression levels and metabolite abundances. We conducted a significance test for these correlation coefficients, calculating P-values using either the t-distribution or the normal distribution. To visualize the co-expression network following the screening process, we employed cytoscape, an open-source software platform for complex network analysis and visualization. In constructing the network, we applied threshold screening criteria, including only interactions with an absolute Spearman correlation value > 0.8 and a P-value < 0.05. As shown in Fig 5, this co-expression network analysis plays a pivotal role in understanding the complex interactions between differentially expressed proteins and metabolites. The network reveals significant positive and negative correlations between various organic acids and their derivatives with CTSS, a protein implicated in the pathogenesis of DKD. Specifically, we observed that certain metabolites, such as 2,4-dihydroxy-3,5,6-trimethylbenzoic acid and 6-phenyl-1-(4-pyridinylmethyl)-1H-pyrazolo[3,4-b]pyridine-4-carboxylic acid, were positively correlated with CTSS. This suggests a potential exacerbating effect on DKD progression under HFD conditions. On the other hand, the negative correlation of peptide fragments like Lys, Ile, and Glu, with CTSS under HFD+ADF conditions may indicate a protective or mitigating role against DKD progression. These findings highlight the differential impact of the dietary interventions on the molecular signatures associated with DKD, providing novel insights into the mechanisms by which ADF may modulate the metabolic landscape and potentially ameliorate DKD outcomes.

### MADF might protect diabetic kidneys against DKD by inhibiting CTSS

**Fig 6:**
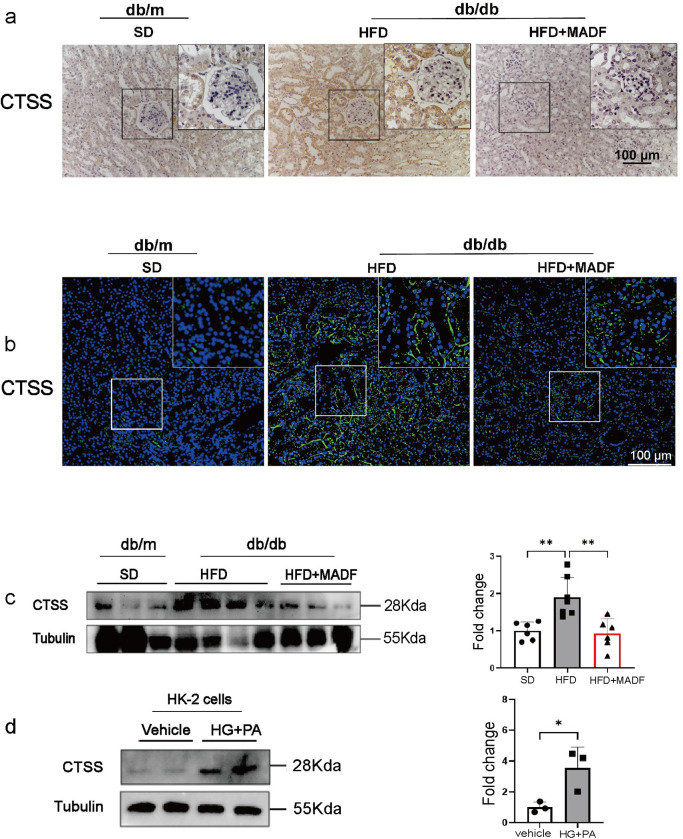
CTSS expression in renal tissues and in HK-2 cells in high glucose and palmitic acid. (a) Immunohistochemical staining of CTSS in mouse renal tissues in the three groups. (b) Immunofluorescence staining of CTSS in mouse renal tissues in the three groups. (c) (d) Representative western blotting analysis for CTSS in renal tissues (SD group*, *n** =  6; HFD group, *n* =  7; and HFD +  MADF group, *n* =  6) and HK-2 cells treated with high glucose and palmitic acid. One-way ANOVA and Tukey’s HSD test were used to analyze the data in (c). And student’s t-test was used to analyze the data in (d). Data are expressed as means ±  standard deviations. * P <  0.05, **P <  0.01, ***P <  0.001.

Considering the notable modifications in CTSS, CTSD, and CTSA observed after the MADF intervention and the effect of CTSS on DKD reported by prior research, we hypothesized that MADF could mitigate DKD by suppressing CTSS expression. Therefore, we verified this in renal tissues and HK-2 cells and found that the expression of CTSS increased in the renal tissues of DKD mice and significantly decreased after the MADF intervention (Fig 6a–6b) (renal tissue: one-way ANOVA: F =  11.57, *P* =  0.001. Tukey’s HSD test: SD vs. HFD, MD =  -0.908, 95% CI =  -1.508 to -0.3085, *P* =  0.0034, t-statistic =  -3.91; SD vs. HFD +  MADF, MD =  0.08946, 95% CI =  -0.5327 to 0.7116, *P* =  0.9273, t-statistic =  -0.37; HFD vs. HFD +  MADF, MD =  0.9957, 95% CI =  0.3979 to 1.597, *P* =  0.0015, t-statistic =  4.29) (HK-2 cells: student’s *t*-test: MD =  2.565, 95% CI =  0.3713 to 4.758, *P* =  0.00315). Subsequently, we evaluated the intracellular CTSS changes in an environment with HGPA using HK-2 cells and found that CTSS expression in HK-2 cells increased after treatment with HGPA.

## Discussion

DKD is a common microvascular complication among individuals with diabetes that reduces their quality of life and increases mortality. While clinical treatment modalities for DKD are limited, dietary restriction has recently been recommended as a nonpharmacological treatment modality for DKD [48]. ADF has become a popular dietary restriction regimen in recent years, and previous studies have demonstrated its effectiveness in reducing body weight, waist circumference, and lipid levels in overweight populations [30,49]. ADF has also been reported to improve DKD in mouse models of type 1 and type 2 DKD [33,50]. In the current study, we investigated whether MADF ameliorated DKD and explored its possible mechanisms of action. We established a type 2 DKD model using db/db mice. Consistent with the results of previous animal experiments [33,50], we found that elevated UACR and pathological progression of DKD were effectively alleviated by MADF. While hyperglycemia is still prevalent in HFD+MADF groups, the mean blood glucose levels were notably lower in the HFD+MADF group compared to HFD group, indicating a potential beneficial effect of MADF on glycemic control. However, we detected random glucose levels in this study. It’s important to consider that variations in hormone levels and feeding times could significantly influence these results, along with potential errors from the glucometer itself. To address these potential sources of variability, we implemented a standardized procedure that included conducting blood glucose tests at the same time point every two weeks and using the same glucometer and a uniform brand of test strips throughout the study. Despite our efforts to minimize errors through standardized operations and equipment, we acknowledge that there are still potential sources of error that may affect our results. The renal proteomic results indicated that protein expression in the kidneys of mice with DKD was altered after the MADF intervention, with more pronounced changes in lysosomal enzymes. The expression of CTSS, CTSA, and CTSD significantly increased in mice with DKD but decreased after the MADF intervention. Subsequently, we verified the changes in CTSS using HK-2 cells in an environment with HG PA. Our cellular experiments showed that the CTSS expression increased in HK-2 cells in an environment with HGPA.

ADF, one of the most studied types of intermittent fasting, effectively ameliorates metabolic diseases. Clinical evidence has shown that ADF can effectively help overweight people in losing weight, reducing their body mass index, blood lipid levels, and blood pressure, and providing cardiovascular protection and is therefore better than other types of intermittent fasting [30,32,49,51,52]. ADF also reduces blood glucose levels, increases insulin sensitivity, improves insulin resistance, and effectively alleviates type 2 diabetes mellitus in humans [53–55]. In one animal study, ADF can improve metabolic disorders in mice with type 1 DKD, improve renal function indexes, reduce urinary protein excretion, and delay the progression of DKD. This study demonstrated that the protective effect of ADF against DKD was achieved by decreasing p53 and increasing Sir2 expression [50]. Our results, on the other hand, suggest that the protective effect of MADF on an animal model of type 2 DKD may be achieved by inhibiting CTSS expression. Mouse renal proteomic results analysis showed that CTSS expression was significantly elevated in renal tissues in the DKD model constructed in db/db mice and was reduced after MADF intervention. Our study demonstrates that in a type 2 DKD animal model, the protective effect of ADF against DKD is associated with the inhibition of CTSS expression. It is important to note that this finding may not be applicable to type 1 DKD, where ADF has been reported to modulate p53 and Sir2 expression, indicating distinct mechanisms of action between the two types of diabetes. The differential mechanisms underscore the complexity of diabetes management and suggest that therapeutic strategies may need to be tailored to the specific type of diabetes. Stephanie et al. showed that ADF effectively reduced the blood glucose and blood lipid levels, 24-h urine output, and renal weight in mice with type 2 DKD but did not effectively reduce the UACR [33]. Our results suggest that MADF improves blood glucose levels, reduces the UACR, improves the DKD phenotype, and exerts a protective effect against DKD. The different effects on the UACR may be due to the different amounts of diet provided on the “fasting days” and the different duration of the intervention. In Stephanie et al.‘s study, the “fasting day” provided 1/2 of the diet on the “eating day,” whereas we provided only 1/3. In addition, the total intervention duration for our MADF was three months, whereas Stephanie et al. provided a 2-month intervention.

Cathepsins are proteases found in the cells of various animal tissues, especially in lysosomes. They are classified according to their active site residues as aspartic proteases (tissue proteases D and E), serine proteases (tissue proteases A and G), and cysteine proteases (tissue proteases B, C, F, H, K, L, O, S, V, W, and X) [56]. Cathepsins are closely associated with various metabolic diseases, including diabetic complications, and are a class of target proteases that have received considerable attention in recent years. Increased CTSS expression in the serum of elderly people correlate with diminished insulin responsiveness and a heightened risk of type 2 diabetes mellitus [57]. CTSA and cathepsin B (CTSB) are capable of worsening diabetic cardiomyopathy and exacerbating cardiac impairment [58,59]. CTSB and CTSD are instrumental in the progression of diabetic retinopathy, highlighting their crucial roles in its worsening condition [60,61]. High levels of cathepsin H are associated with impaired pancreatic β-cell function [62]. Urinary CTSD is associated with a rapid decline in the estimated glomerular filtration rate (eGFR) and an increase in urinary protein levels in patients with type 1 diabetes and is an effective biological indicator for predicting the progression of DKD [63]. These studies suggest that CTS is an essential target in diabetes. As illustrated in Fig 3c, levels of CTSA, CTSD, and CTSS levels were elevated in DKD mice. Kumar et al. demonstrated that serum CTSS levels positively correlated with blood creatinine and urea nitrogen levels and negatively correlated with eGFR, suggesting that CTSS was significantly associated with CKD progression. The CTSS/PAR2 pathway triggers glomerular endothelial cell dysfunction and promotes DKD development [64]. In our study, we found that MADF reduced the elevated CTSS levels in DKD, suggesting that the protective effect of MADF on DKD may be realized by inhibiting CTSS expression. These results were verified in cellular experiments, in which CTSS was elevated in HK-2 cells in an environment with HGPA. Another study showed that CTSS is closely associated with diabetic complications such as insulin resistance, impaired glucose tolerance, and atherosclerosis [65]. Therefore, CTSS emerges as a promising candidate for a new therapeutic approach in the treatment of DKD.

Our study has some limitations. First, the effect of MADF on DKD was mainly assessed using UACR values in our experiments. Urea nitrogen, creatinine, eGFR, and other indices were not measured to evaluate renal function. Second, we did not perform gene knockdown or use specific inhibitors in animals to verify the effect of CTSS inhibition on DKD. Further research is required to evaluate the ameliorative effect of MADF in patients diagnosed with DKD.

## Conclusions

This is the first study to explore the possible mechanism by which MADF could ameliorate DKD using proteomic and metabolomic linkage analyses. Our results suggested that MADF was effective in halting DKD progression. The proteomic analysis highlighted lysosome-related proteins, notably CTSS, as crucial mediators of the renoprotective effects of MADF in DKD. MADF might exert its ameliorative effect on DKD via inhibition of CTSS expression. This research highlights the utility of MADF and CTSS inhibition as promising therapeutic strategies for managing DKD.

## Supplementary Information

S1 Fig Linear regression analysis of mice body weight. (TIF)

S1 Table In comparison to the control group, differentially expressed proteins in the kidneys of DKD mice. (XLSX)

S2 Table After MADF intervention, differentially expressed proteins in the kidneys of DKD mice. (XLSX)

S3 Table In comparison to the control group, differentially expressed metabolites in the kidneys of DKD mice. (XLSX)

S4 Table After MADF intervention, differentially expressed metabolites in the kidneys of DKD mice. (XLSX)

S1 Raw Images The original western blot images involved in the study. (PDF)
