# Supplementary figures and images for: Proteomic and metabolomic profiling reveals the underlying molecular mechanisms in modified alternate-day fasting-mediated protection against Diabetic kidney disease
Source: PLoS One. 2025 Feb 18;20(2):e0319053. doi: 10.1371/journal.pone.0319053 (PMC11835337; doi:10.1371/journal.pone.0319053)

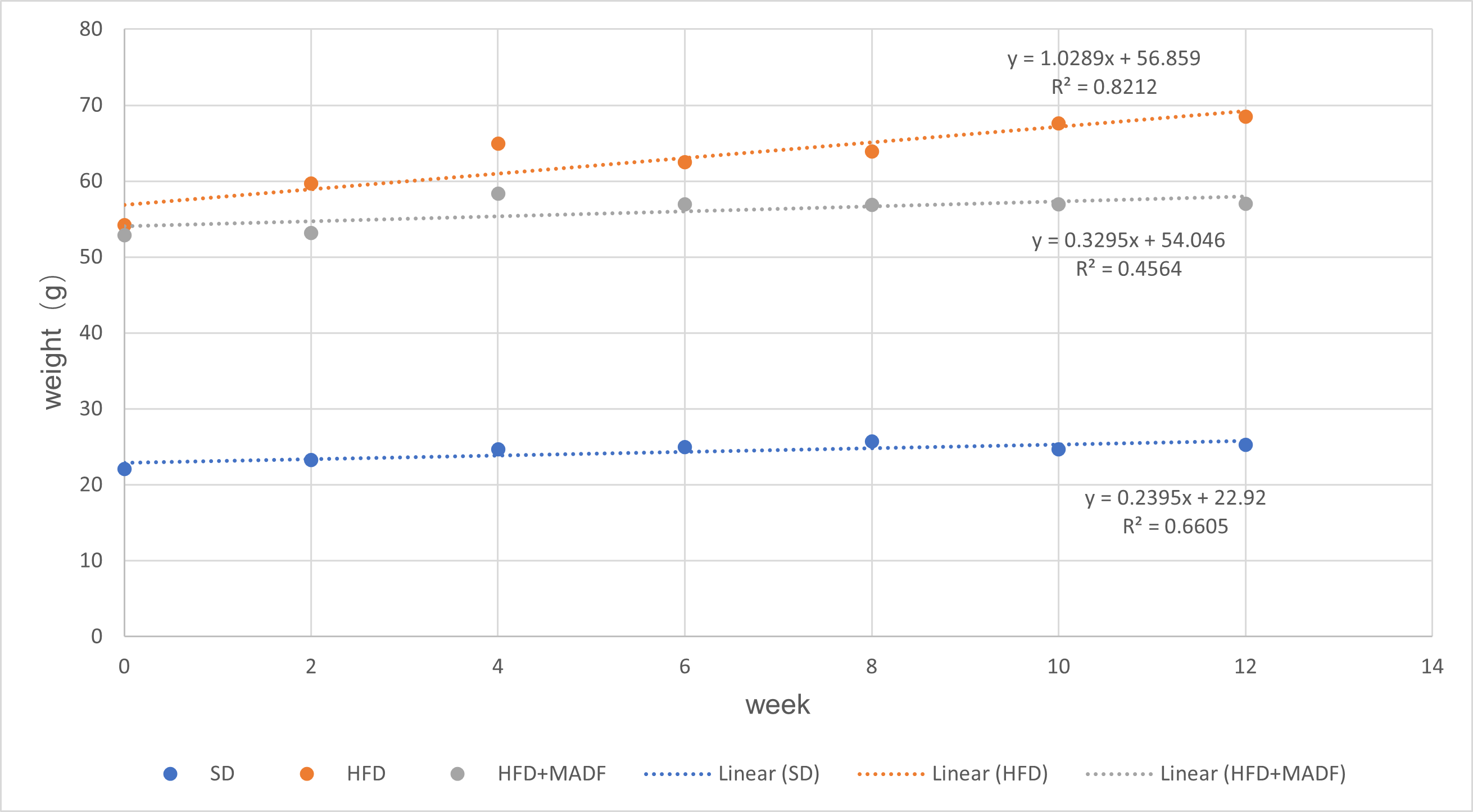

Supplement: S1 Fig — (TIF) [file pone.0319053.s001.tif]
