# Supplementary material for: Proteomic and metabolomic profiling reveals the underlying molecular mechanisms in modified alternate-day fasting-mediated protection against Diabetic kidney disease
Source: PLoS One. 2025 Feb 18;20(2):e0319053. doi: 10.1371/journal.pone.0319053 (PMC11835337; doi:10.1371/journal.pone.0319053)
Supplement: S1 Raw Images — (PDF) [file pone.0319053.s006.pdf]

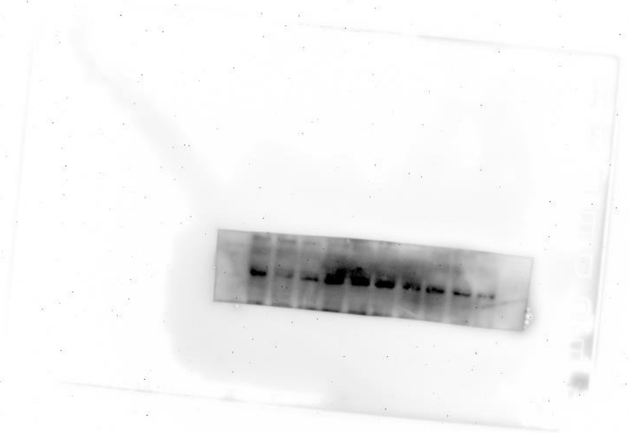

Original western blot image of Cathepsin S (CTSS) in kidney tissue, corresponding to Figure 6c.

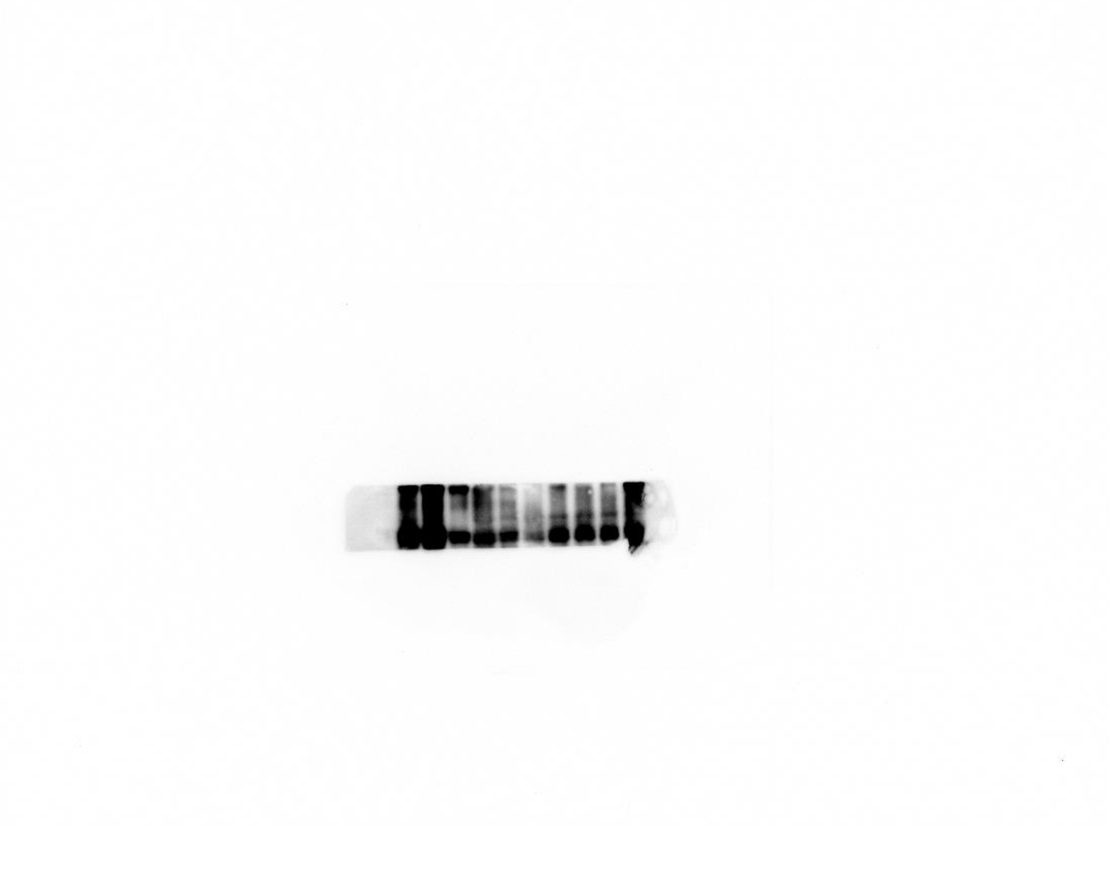

Original western blot image of the marker tubulin for Figure 6c.

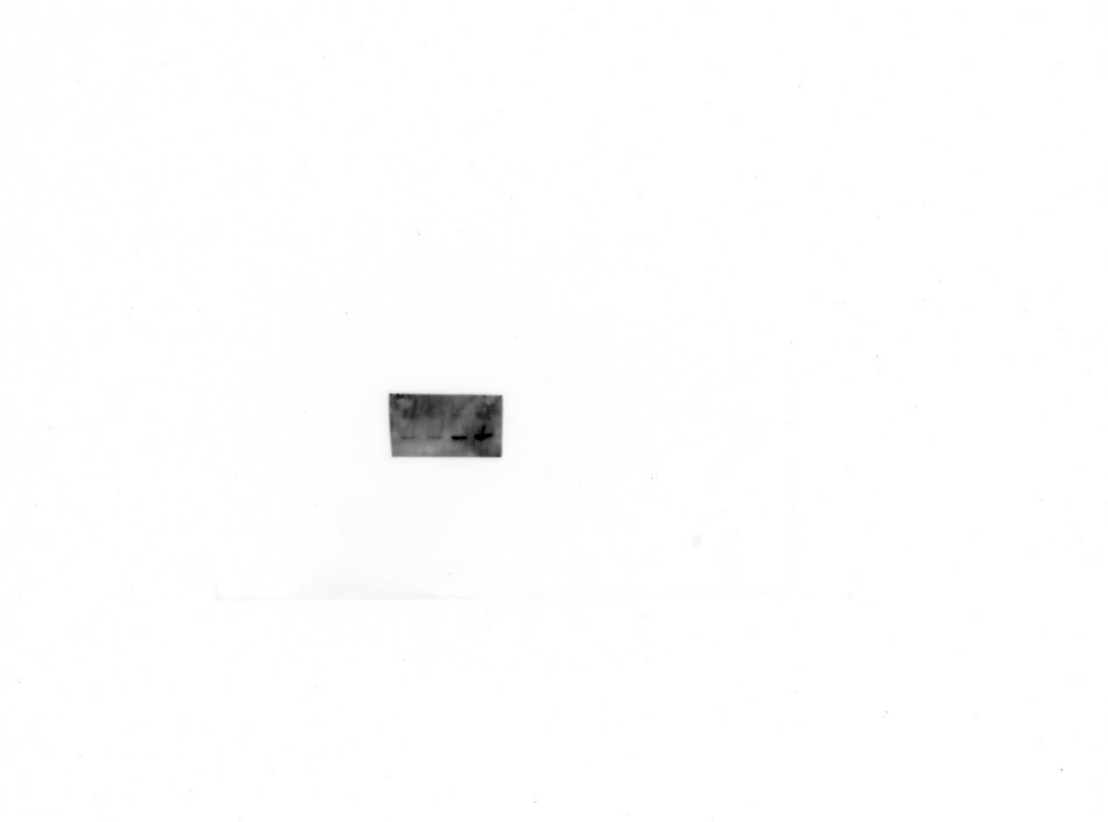

Original western blot image of CTSS in HK-2 cells, corresponding to Figure 6d.

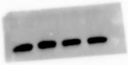

Original western blot image of the marker tubulin for Figure 6d.
